# Effect of Recombinant NGF Encapsulated in Chitosan on Rabbit Sperm Traits and Main Metabolic Pathways

**DOI:** 10.3390/biology14080974

**Published:** 2025-08-01

**Authors:** Luigia Bosa, Simona Mattioli, Anna Maria Stabile, Desirée Bartolini, Alessia Tognoloni, Alessandra Pistilli, Mariangela Ruggirello, Mario Rende, Silvia Gimeno-Martos, Daniela Jordán-Rodríguez, Maria Arias-Álvarez, Pilar García Rebollar, Rosa M. García-García, Cesare Castellini

**Affiliations:** 1 Department of Agricultural, Food and Environmental Sciences, University of Perugia, 06121 Perugia, Italy; simona.mattioli@unipg.it (S.M.); cesare.castellini@unipg.it (C.C.); 2 Department of Medicine and Surgery, University of Perugia, 06129 Perugia, Italy; anna.stabile@unipg.it (A.M.S.); alessandra.pistilli@unipg.it (A.P.); mario.rende@unipg.it (M.R.); 3 Department of Pharmaceutical Sciences, University of Perugia, 06122 Perugia, Italy; desiree.bartolini@unipg.it (D.B.); alessia.tognoloni@collaboratori.unipg.it (A.T.); 4 Department of Translational Medicine and Surgery, Section of General Pathology, Università Cattolica del Sacro Cuore, Largo Francesco Vito 1, 00168 Rome, Italy; mariangela.ruggirello@unicatt.it; 5 Department of Animal Physiology, Faculty of Veterinary Medicine, Universidad Complutense de Madrid, 28040 Madrid, Spain; silvigim@ucm.es (S.G.-M.); djordan@ucm.es (D.J.-R.); m.arias@vet.ucm.es (M.A.-Á.); rosa.garcia@vet.ucm.es (R.M.G.-G.); 6 Department of Animal Production, School of Agricultural, Food and Biosystems Engineering, Polytechnic University of Madrid, 28040 Madrid, Spain; pilar.grebollar@upm.es

**Keywords:** apoptosis, acrosome reaction, capacitation, rabbit sperm, signaling cascades

## Abstract

The aim of this study was to analyze how recombinant rabbit NGF (Nerve Growth Factor) encapsulated in chitosan (rrβNGFch) affects sperm viability, motility, capacitation, acrosome reaction (AR), kinetic traits, and apoptosis after 30 min and 2 h of storage. Specific intracellular signaling pathways associated with either cell survival, such as protein kinase B (AKT) and extracellular signal-regulated kinases 1/2 (ERK1/2), or programmed cell death, such as c-Jun N-terminal kinase (JNK), were also analyzed. The results confirmed the effect of rrβNGFch on capacitation and AR, whereas a longer storage time (2 h) decreased all qualitative sperm traits. AKT and JNK did not show treatment-dependent activation and lacked a correlation with functional traits, as shown by ERK1/2. These findings suggest that rrβNGFch may promote the functional activation of sperm cells, particularly during early incubation. The increase in capacitation and AR was not linked to significant changes in pathways related to cell survival or death, indicating a specific action of the treatment. In contrast, prolonged storage negatively affected all sperm parameters. ERK1/2 activation correlated with capacitation, AR, and apoptosis, supporting its role as an NGF downstream mediator. Further studies should analyze other molecular mechanisms of sperm and the potential applications of NGF in assisted reproduction.

## Simple Summary

Nerve Growth Factor (NGF) is present in rabbit seminal plasma and plays a role in sperm function. In this study, we investigated the effect of NGF encapsulated in chitosan microspheres (rrβNGFch) on rabbit sperm stored at room temperature for 30 min or 2 h. The encapsulated NGF improved sperm activation, specifically the capacitation and acrosome reaction after 30 min. However, storage for 2 h negatively affected the overall sperm quality. Among the intracellular pathways analyzed, only ERK1/2 was significantly activated by rrβNGFch and correlated with functional traits. These results highlight the potential usefulness of NGF microencapsulation for improving the sperm quality in reproductive technologies.

## 1. Introduction

The Nerve Growth Factor (NGF) is a neurotrophin involved in the regulation of neuronal survival and differentiation [1]. Numerous studies have demonstrated the widespread distribution of NGF across various tissues including the reproductive tract [2]. The involvement of NGF in modulating the essential traits of mature sperm has been reported in multiple species [3,4,5,6,7].

NGF in male reproductive secretions has been detected in the prostate of many animal species, such as guinea pigs, rabbits, pigs, and bulls [8], Harper and Thoenen, 1980 [9,10]. NGF has also been detected in the seminal plasma of various other livestock species, including bulls [11,12,13], rabbits, camels, llamas, and alpacas, which have become the focus of extensive research [14,15,16]. Accordingly, NGF and its receptors TrkA and p75 are broadly expressed in the testis, accessory reproductive glands, and epididymal spermatozoa [9]. NGF may trigger several processes mediated by two receptors, tropomyosin receptor kinase A of 140-kDa (TrkA), and the NGF receptor of 75 kDa (p75NTR), of a high and low affinity, respectively [17,18].

In rabbit seminal plasma, NGF is mainly secreted by the prostate and seminal vesicles and can bind receptors on spermatozoa of different species, thereby influencing their functions [19]. In particular, NGF plays a key role in regulating the survival and senescence of spermatozoa, depending on which receptor is activated: the pro-apoptotic p75NTR or the pro-survival TrkA pathways. NGF-TrkA activation promotes sperm survival and regulates the acrosome reaction (AR) via kinase signaling pathways (mitogenic p38-mitogen-activated protein kinase, MAPK, and pro-survival phosphatidylinositol-3-kinase/protein kinase B PI3K/AKT). In contrast, NGF binding to p75NTR influences sperm apoptosis, motility, and respiratory chain function [20] and induces apoptosis via Jun N-terminal Kinase (JNK)/caspases or survival via a nuclear factor kappa light chain enhancer of activated B cells and extracellular signal-regulated kinases (ERK1/2).

NGF, along with its high- and low-affinity receptors (TrkA and p75NTR, respectively), is also present in all compartments of the rabbit ovary and oviduct, suggesting a physiological role in ovarian folliculogenesis, steroidogenesis, ovulation, and luteogenesis [2]. NGF has also been investigated for its potential role in inducing ovulation in female rabbits [21]. In rabbits, ovulation occurs normally in response to mating. However, in artificial insemination (AI), this natural stimulus is typically replaced by the administration of the exogenous gonadotropin-releasing hormone (GnRH) to trigger ovulation. Recent studies have proposed that seminal NGF is a physiological inducer of ovulation [22]. Some evidence suggests that seminal NGF, whether delivered alone or in conjunction with the mechanical stimulation of insemination (e.g., the use of a cannula), influences the hormonal cascade involved in ovulation. Additionally, intramuscular, intravenous, or intravaginal NGF administration has been shown to modulate LH secretion in response to GnRH stimulation [21]. It should be noted that seminal plasma has a high level of aminopeptidase activity [23,24] that rapidly degrades NGF added to the seminal dose before the vaginal epithelium can fully absorb it, thereby diminishing its bioavailability [25].

Microencapsulation is widely employed to prevent enzyme activity on specific molecules, modulate drug release over time, and optimize the uptake of epithelial molecules’ uptake [22,26]. Chitosan, a deacetylated derivative of chitin, is one of the most used polymers for microencapsulation in the controlled release of various bioactive compounds [27].

NGF demonstrated enhanced effectiveness when encapsulated in chitosan-based delivery systems. Sanchez-Rodriguez et al. [28] demonstrated that encapsulating NGF in chitosan prolongs its release, potentially enhancing its physiological action and favoring ovulation. Quiroga et al. [22] showed that chitosan microspheres added to semen did not affect the rabbit sperm quality; therefore, they could be included in the seminal doses for AI. Moreover, the same authors showed that microencapsulated recombinant rabbit βNGF (rrβNGFch) did not negatively affect sperm traits or induce capacitation and AR during in vitro incubation. In another study, an NGF addition confirmed its effect on AR [29], showing associated changes due to the capacitation process and modulation by p75NTR [18,29]. Consequently, encapsulation is crucial for maintaining the role of NGF in sperm and, at the same time, for improving the ovulation process in rabbit does.

Given the role of NGF in modulating key sperm processes, this study aimed to understand how NGF affects the sperm viability, motility, and overall physiological competence. In recent years, several studies have aimed at improving the formulation of semen extenders by including functional additives such as amino acids, antioxidants, and plant-based compounds, with the goal of preserving the sperm viability and enhancing the fertilizing potential. Among these, L-arginine has been reported to improve the post-thaw semen quality and fertility in rams subjected to transcervical artificial insemination. Similar strategies have also been explored in rabbits, suggesting the value of supplementing extenders to support the sperm function during storage and manipulation. These approaches reflect a growing interest in the practical optimization of semen preservation protocols for assisted reproduction in different species [22].

Special attention is given to the analysis of specific intracellular signaling cascades that are indicative of either cell survival (AKT and ERK1/2) and/or programmed cell death (JNK). By comparing the effects of endogenous and chitosan-encapsulated NGF, this study also aimed to determine whether sustained-release formulations affect these signaling responses.

## 2. Materials and Methods

If not otherwise specified, all chemicals were purchased from Sigma-Aldrich (St. Louis, MO, USA). RrβNGF, corresponding to the gene number KX528686, was produced as described by Sanchez-Rodriguez et al. [28] and was added to the seminal doses. Microencapsulation of rrβNGF was performed according to previously established protocols [22,30]. Microspheres with a diameter smaller than 10 µm were obtained, containing between 0.24 and 0.44 µg of rrβNGF per mg of microspheres. Prior to use, lyophilized rrβNGF microspheres (rrβNGFch) and the corresponding solution of chitosan microspheres were suspended in sterile filtered autoclaved in phosphate-buffered saline (PBS). 

### 2.1. Animal Model

Eight adult New Zealand White rabbit bucks (8 months of age) were raised on the experimental farm of the Department of Agriculture, Food, and Environmental Science of Perugia (Italy) and used for semen collection. The rabbit bucks were healthy, previously trained for semen collection, and confirmed to be fertile based on prior assessments. Semen collection was performed once per week by using a dummy and an artificial glass vagina maintained at internal temperature of 37 °C.

Specific guidelines for rabbit bucks and the International Guiding Principles for Biomedical Research Involving Animals [31] were followed in this study. The animals were reared in compliance with the 2010/63/EU directive. The experiment did not require specific authorization by the ethical committee because the animals were not subjected to stressful treatment, causing pain and suffering.

Three semen collections, every 2 weeks, were performed between May and July 2024.

### 2.2. Semen Handling

After semen collection, samples were evaluated macroscopically (color, presence of urine or feces, and pH); all semen samples exhibited suitable macroscopic characteristics and were, therefore, processed. Sperm traits (capacitation and AR, kinetic traits, fluorescence-activated cell sorting (FACS)scananalysis, and immunoblot assays) were analyzed after 30 and 2 h of storage.

Sperm concentration was measured using a cell-counting chamber and a light microscope (Olympus CH2, Olympus Corporation, Tokyo, Japan) set at 40× magnification.

For each collection, an aliquot of each semen sample (concentration > 300 × 10^6^ cells/mL and motility rate > 75%) was stored for Western blot analysis, while the rest was diluted with a PBS solution supplemented with 0.5% bovine serum albumin (BSA) to achieve a final concentration of 10^6^ sperm/mL. The osmolarity and pH of the samples were 296 mOsm/kg and 7.4, respectively.

The following treatments were tested:

- Control (Ctrl).
- Chitosan (ch).
- NGF + chitosan (rrβNGFch).

Specifically, 1 µg/mL rrβNGFch resuspended in sterile PBS was used according to previous studies [12]. Additionally, an equivalent solution of empty chitosan microspheres (chitosan 3.5 × 10^6^ microspheres in 100 μL PBS) without rrβNGF was added to the seminal doses. A control group, consisting of the same diluted semen without any treatment, was incubated under the same conditions.

### 2.3. Capacitation and Acrosome Reaction of Sperm

The capacitation and acrosome state of rabbit sperm were evaluated using the chlortetracycline fluorescence assay, following the protocol of Gimeno-Martos et al. [32]. Spermatozoa were classified into one of the following patterns: non-capacitated with regular distribution of fluorescence on the head, capacitated with fluorescence in the acrosome, and acrosome-reacted (AR) cells that did not show fluorescence in the head or equatorial region.

### 2.4. Evaluation of Motility and Kinematic Traits

Motility parameters were evaluated using a computer-assisted semen analyzer (model ISAS, Valencia, Spain), according to previously assessed parameters. In brief, the main set up values were the following: frame rate 100 Hz, 12–200; low-velocity cut-off (μm/s): 20 medium-velocity cut-offs (μm/s): 40; progressive linearity cut-off (μm/s): 60; static head size (μm^2^): min 9.5–max 23; and calculation of kinetic traits: >70% tracked points.

Samples were further diluted with PBS to a concentration of 5 × 10^6^ cells/mL and were placed in pre-slide holder at 37 °C. Two drops of each sample and three microscopic fields were analyzed. All kinematic parameters were assessed (i.e., VSL, STR, BCF, and WOB), but only the main indicators, motility rate (% of total motile cells on the total sperm), curvilinear velocity (VCL, μm/s: the velocity of the sperm head along its actual trajectory), linearity (LIN, %: ratio between VSL and VCL), and ALH (μm of lateral head displacement), are reported in the tables.

### 2.5. FACSscan Analysis of TrKA and p75NTR Receptors

Aliquots of sperm were washed thrice in PBS/BSA and centrifuged at 400× *g* for 5 min. Subsequently, aliquots of 1 × 106/mL of sperm were placed in FACSscan tubes and pre-incubated with PBS/BSA for 30 min at 4 °C. Cells were then centrifuged and incubated for 1 h in PBS/BSA containing 2.5 μg/106 cells of anti-TrKA (AF175, R&D System, Minneapolis, MN, USA) and 2 μg/106 cells of anti-p75NTR (MA5–13314, Thermo Fisher Scientific, Waltham, MA, USA) at 4 °C. Afterwards, the cells were washed in PBS/BSA and incubated with the secondary antibodies (0.2 μg/mL ab72465 PE conjugated for TrKA and 2 μg/mL ab6785 FITC conjugated for p75NTR, Abcam, Cambridge, UK) for 30 min at 4 °C.

After incubation, cells were washed and rinsed with PBS/BSA. TrKA-positive and p75NTR-positive cells were quantified by FACS scan analysis using a FACScan Calibur (Becton Dickinson, San Jose, CA, USA). Ten thousand live-gated events were collected for each sample, and isotype-matched antibodies were used to determine the binding specificity. The results are expressed as the percentage of positive cells/antibody used for staining (% positive cells).

### 2.6. Determination of Live, Apoptotic, and Necrotic Sperm

Detection of phosphatidylserine externalization was performed using the Annexin V Apoptosis Detection Kit (K101–100 BioVision, Milpitas, CA, USA), made up of annexin V–fluorescein isothiocyanate (AnV–FITC) and propidium iodide–phycoerythrin (PI-PE), which can differentiate viable from necrotic and apoptotic cells. Aliquots of experimental samples were washed with PBS, centrifuged, and suspended in 500 μL of annexin-binding buffer to obtain a cell count of approximately 1 × 10^5^. Five microliters of AnV–FITC and 5 μL of PI–PE (50 μg/mL) were added to each cell suspension. The samples were incubated at RT for 5 min in the dark and then analyzed by flow cytometry. Flow cytometry analysis was performed with a FACScan Calibur by plotting green fluorescence (FL1)/AnV–FITC vs. red fluorescence (FL2)/PI–PE-positive cells. Flow cytometry data acquisition was performed on a FACScan Calibur equipped with 488 and 633 nm lasers and running the CellQuest Software v5.1(Becton Dickinson, San Jose, CA, USA). Ten thousand events were recorded for each sample.

### 2.7. Protein Extraction and Quantification

Aliquots of rabbit sperm were lysed in ice-cold Cell Lysis Buffer purchased from Cell Signaling Technology (CST) containing 20 mM Tris–HCl (pH 7.5), 150 mM NaCl, 1 mM Na_2_EDTA, 1 mM ethylene-glycol-tetra acetic acid (EGTA), 1% Triton, 2.5 mM sodium pyrophosphate, 1 mM β-glycerophosphate, 1 mM Na_3_VO_4_, 1 μg/mL leupeptin, and 1X Protease Inhibitor Cocktail (200X, #7012, Cell Signaling Technology, Danvers, MA, USA). Debris was removed by centrifugation at 14,000× *g* for 30 min at 4 °C. The BCA Protein Assay (Pierce, Thermo Fisher Scientific Inc., Waltham, MA, USA) was used to quantify total protein concentrations according to the manufacturer’s instructions using bovine serum albumin (BSA, Sigma-Aldrich) as a standard.

### 2.8. Immunoblot Assay

Protein extracts obtained from rabbit sperm (20 μg of total protein) were heat-treated in loading sample buffer containing dithiothreitol (DTT) and processed for protein separation by 4–12% sodium dodecyl sulfate–polyacrylamide gel electrophoresis (SDS-PAGE). Proteins of slab gels were then transferred to nitrocellulose membrane, at 0.45 µm (Bio-Rad Laboratories S.r.l., Segrate, Italy). Membranes were incubated for 5 min in EveryBlot Blocking Buffer (Bio-Rad Laboratories S.r.l.) and then overnight with primary antibodies that included Phospho-AKT (Ser473) (D9E) XP^®^ Rabbit mAb (#4060, 1:1000, Cell Signaling Technology, CST), anti- AKT Antibody (#9272, 1:1000, Cell Signaling Technology, CST), Phospho-p44/42 MAPK (ERK1/2) (Thr202/Tyr204) Antibody (#9101, 1:1000, Cell Signaling Technology, CST), p44/42 MAPK (ERK1/2)(#9102, 1:1000, Cell Signaling Technology, CST anti-Phospho-JNK (T183/Y185) Antibody (#AF1205, 1:1000, R&D Systems), anti- JNK Pan Specific Antibody (#AF1387, 1:1000, R&D Systems), and β-Tubulin Antibody (#2146, 1:1000, Cell Signaling Technology, (CST), Danvers, MA, USA) diluted in EveryBlot Blocking Buffer (Bio-Rad Laboratories S.r.l.) or BSA (Sigma-Aldrich). After three washes with Tris-buffered saline (TBS) containing 0.01% Tween-20, horseradish peroxidase-conjugated anti-mouse (#7076) or anti-rabbit (#7074) secondary antibody (Cell Signaling Technology, CST) diluted by 1:2000 in Every Blot Blocking Buffer was added, and protein bands were detected using a ChemiDoc Imaging System (Bio-Rad Laboratories, Hercules, CA, USA) using an ECL Clarity Max (Bio-Rad Laboratories, Hercules, CA, USA). Band quantification was performed with Image Lab (version 6.1.0, build 7, Standard Edition, Bio-Rad Laboratories Inc.).

### 2.9. Statistical Analysis

The effect of treatment (control, chitosan, and rrβNGFch) and storage time (30 min vs. 2 h) was estimated using a mixed model considering the repeated effect of buck. However, the two-way interaction was not significant. Values were considered statistically significantly different when *p* ≤ 0.05. Pearson’s correlation coefficients were also calculated. Different Stata program procedures were used Stata v14.0 (StataCorp LLC, College Station, TX, USA) [33].

## 3. Results

In this study, we analyzed the expression of TrKA and p75NTR receptors and the activation of the main NGF-induced signaling pathways in rabbit sperm with or without the addition of chitosan spheres alone or rrβNGF encapsulated in chitosan. Furthermore, we evaluated the effects of these additions on the main NGF-induced pathways.

The addition of rrβNGFch and the storage time (30 min vs. 2 h) significantly affected several in vitro sperm traits (Table 1). Specifically, in vitro treatment influenced the proportion of capacitated and acrosome-reacted sperm cells as well as ERK1/2 activation.

Conversely, the storage time affected live and motile sperm, apoptotic and capacitated sperm, P75NTR expression, and the activation of AKT pathways. As expected, after 2 h of storage, live, motile, apoptotic, and capacitated cells decreased in all the experimental groups (*p* < 0.05).

**Figure 1:**
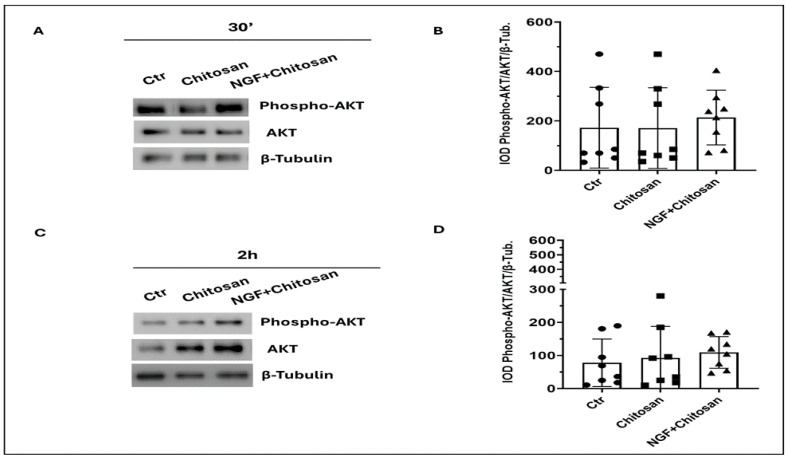
Effect of chitosan (Chitosan) and recombinant rabbit β nerve growth factor encapsulated in chitosan microspheres (rrβNGFch) on AKT activity in rabbit sperm. (**A**) Immunoblot of phosphorylated AKT (Phospho-AKT) and total AKT after 30 min of treatment with chitosan alone or with rrβNGFch. (**B**). Densitometric analysis of Phospho-AKT/AKT/β-Tubulin after 30 min of treatments. (**C**) Immunoblot of Phospho-AKT and total AKT after 2 hrs of treatment with chitosan alone or with rrβNGFch. (**D**). Densitometric analysis of Phospho AKT/AKT/β-Tubulin after 2 h. 30′ vs. 2 h. Significant differences between groups were detected (*p* < 0.05); values are shown in the table. The corresponding full-length Western blots are shown in Supplementary Materials.

**Figure 2:**
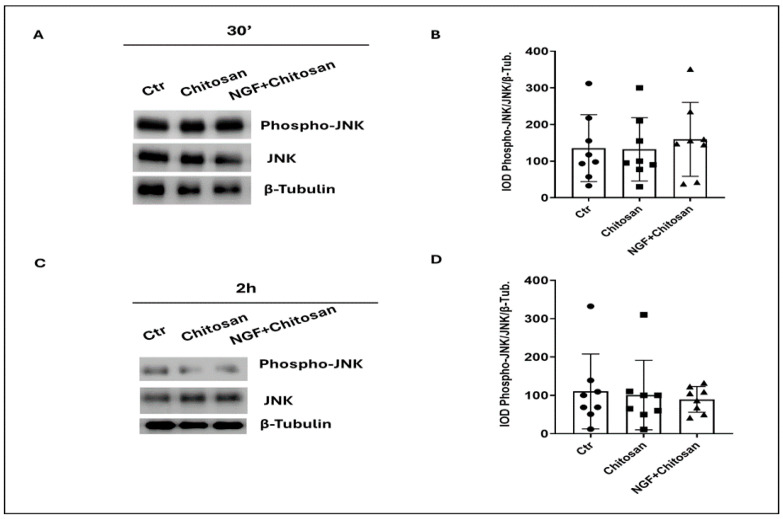
Effect of chitosan and NGF + chitosan on JNK phosphorylation in rabbit sperm traits. (**A**) Immunoblot of Phospho-JNK and total JNK after 30 min of treatment with chitosan alone or with rrβNGFch. (**B**). Densitometric analysis of Phospho-JNK/JNK/β-Tubulin after 30 min of treatments. (**C**) Immunoblot of Phospho-JNK and total JNK after 2 h of treatment with chitosan alone or with rrβNGFch. (**D**) Densitometric analysis of Phospho JNK/JNK/β-Tubulin after 2 h. The corresponding full-length Western blots are shown in Supplementary Materials.

Figure 1 and Figure 2 showed the main NGF-derived pathways with or without the addition of rrβNGFch and chitosan spheres. As shown in Figure 1, AKT activation seemed to be mainly influenced by the time of storage and not by the addition rrβNGFch or chitosan even if a tendency (*p* = 0.06) towards AKT activation was observed following treatment with rrβNGFch. The lack of significance was probably due to high inter-individual variability (Figure 1B,D).

Additionally, in Figure 2, the mild activation of JNK was observed after 30 min of incubation with rrβNGFch (Figure 2A,B), followed by a tendency (*p* = 0.08) towards a decrease after 2 h of treatment with rrβNGFch (Figure 2C,D). 

**Figure 3:**
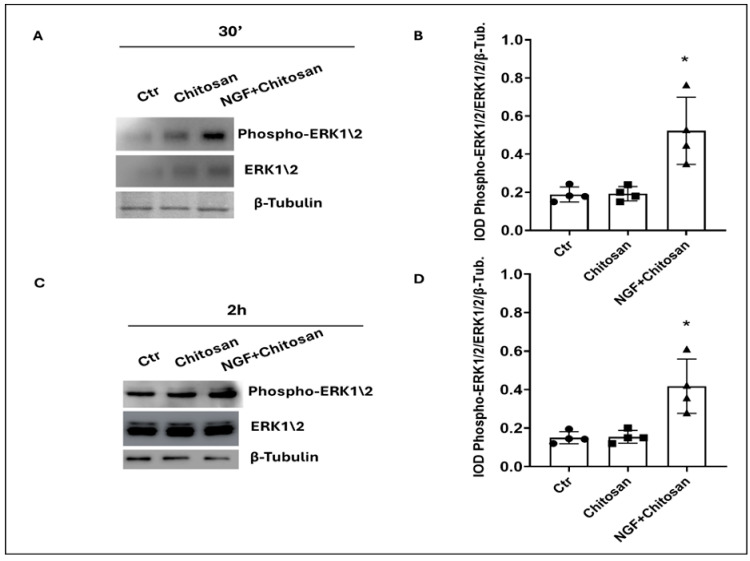
Effect of chitosan and NGF + chitosan on ERK1/2 phosphorylation in rabbit sperm traits. (**A**) Immunoblot of Phospho-ERK1/2 and total ERK1/2 after 30 min of treatment with chitosan alone or with rrβNGFch. (**B**) Densitometric analysis of Phospho-ERK1\2/ERK1\2/β-Tubulin after 30 min of treatments. (**C**) Immunoblot of Phospho-ERK1/2 and total ERK1/2 after 2 h of treatment with chitosan alone or with rrβNGFch. (**D**) Densitometric analysis of Phospho ERK1/2/ERK1/2/β-Tubulin after 2 h. *t*-test: Ctr vs. NGF, * *p* < 0.05. The corresponding full-length Western blots are shown in Supplementary Materials.

In contrast, the activation of ERK1/2 by rrβNGFch was clearly showed in Figure 3 (*p* < 0.05). The full-length blots corresponding to these experiments are provided in Supplementary Materials.

The correlation coefficients (Table 2) showed positive values for ERK1/2, capacitation (*p* < 0.05), AR (*p* < 0.01), and apoptosis (*p* < 0.01). Similarly, p75NTR, was positively correlated with relevant sperm traits (capacitation and AR) (*p* < 0.05) and apoptosis (*p* < 0.01). 

## 4. Discussion

The role of NGF and its receptors in spermatogenesis has been previously evaluated in several animal species and in humans [29,34,35,36]. Other scientific evidence assessed that NGF also modulates key functions in mature sperm, including capacitation, acrosome reaction, apoptosis, the motility rate, and kinetics traits [28,29].

In the present study, we showed that NGF, encapsulated in chitosan or chitosan spheres alone, did not affect the kinetic parameters of sperm (motile cells, VCL, LIN, and ALH). It is widely known that in mammal sperm, NGF exerts its function by binding to two receptors, TrkA and p75NTR. Castellini et al. [29] demonstrated that the effect of NGF on rabbit sperm was modulated by the asset of these cell receptors. While TrKA were almost stable in different samples and during sperm storage, semen outcomes and the trend of the senescence of sperm was affected by variations in p75NTR. Consequently, the balance between “death–survival” pathways, triggered by NGF, seems to be regulated by the p75NTR/TrkA ratio [36,37]. In this context, it should be mentioned that the sperm receptor asset [36] of the present samples, which was close to the threshold value previously defined to distinguish the normal vs. anomalous P75NTR profile, could have influenced the sperm kinetic response.

At the same time, this study confirmed that apoptosis, capacitation, and AR were affected by the NGF-p75NTR interaction. Probably, the higher sperm reactiveness of rrβNGFch samples were also affected by the encapsulation of NGF and by its prolonged release. Light differences in capacitation and AR obtained by Gimeno-Martos et al. [32] could be explained by differences in the baseline characteristics of semen. The encapsulation of NGF, by modulating the availability of the molecule, also improves the triggering of ovulation in the artificial insemination procedure. This technique, avoiding the intramuscular inoculation of GnRH, has garnered interest in the field because is more welfare-oriented [22]. Encapsulated NGF retains its activity on gonadotrophin release and may act via signaling pathways, at least partially independent of those regulating the motility.

Although some interactions between NGF and the metabolic response of in vitro-cultured sperm have been clarified [24,38], the intracellular signaling cascades involved remain largely undefined. NGF binding with TrKA mediates proliferation, differentiation, and survival via the activation of the PI3K/AKT, Ras/ERK/MAPK, and PLCγ pathways. On the contrary, the binding of NGF to p75NTR leads to the activation of NF-kB and JNK reduces survival and mediates apoptosis [37].

Unfortunately, in this study, we did not find any significant activation of AKT or JNK by rrβNGFch and no substantial relationship between AKT or JNK and functional sperm traits (e.g., apoptosis and motility). Accordingly, only partial conclusive considerations can be drawn. In particular, it should be clarified whether this lack of correlation is related to a specific cell type (sperm vs. somatic cells) or to other intrinsic regulatory mechanisms. Previous results in epididymal mouse sperm [38] showed a positive relationship between the phosphorylation of the p-AKT (Thr308) capacitation status, acrosome reaction, and various kinematic parameters. The differences in the sperm collection between studies (mature sperm vs. epididymal sperm) and the experimental procedures used could explain the discrepancies obtained.

At the same time, rrβNGFch appeared to exert a cytoprotective effect via the selective activation of the MAPK ERK1/2 pathway. Jaldety et al. [39] showed that ERK1/2 plays a crucial role in mediating capacitation and AR in mouse sperm. The inhibition of ERK1/2 almost completely abolished the ability of sperm to undergo AR, highlighting its central role in this process. Capacitation is a fundamental prerequisite for both AR and fertilization. The increased phosphorylation and activation of ERK1/2 have been established as critical events leading to intracellular Ca^2+^ elevation, which in turn triggers AR [39]. Volenté et al. [40] confirmed that NGF stimulates the catalytic activity of ERK1 and ERK2 associated with the p75NTR receptor, further supporting a link between NGF signaling and ERK regulation.

Additionally, the storage time affected key sperm parameters, such as the viability, motility rate, apoptosis, and capacitation, highlighting the intrinsic sensitivity of spermatozoa to storage. These time-dependent effects may overlap or mask potential treatment-induced changes in signaling pathways, particularly for AKT and JNK activation, which did not show as clearly treatment-dependent, as shown by ERK ½. The lack of strong correlations between AKT, JNK, and the functional traits suggests a minor role in NGF-mediated responses under our experimental conditions. JNK and AKT activation appeared inconsistent and may reflect a more transient or delayed response that was not fully captured within the analyzed time points. Further studies are warranted to confirm or refute these findings and fully elucidate the intracellular mechanisms underlying NGF action on spermatozoa.

## 5. Conclusions

This study confirmed the positive effect of rrβNGF encapsulated in chitosan microspheres on rabbit sperm capacitation and acrosome reactions, especially after short-term storage. The activation of ERK1/2 was significantly associated with these traits, supporting its role as a mediator of NGF action. Conversely, AKT and JNK pathways were not influenced by the treatment and did not correlate with the sperm functional characteristics. The encapsulation technique appeared to preserve NGF bioactivity, avoiding cytotoxic effects. Despite the lack of a clear intracellular response for some pathways, the results highlight the potential of rrβNGFch as a tool to improve sperm function in reproductive protocols. However, further studies are needed to better understand the molecular mechanisms involved and to assess its practical application in rabbit semen preservation.

## Figures and Tables

**Table 1:** Effect of treatment and time of storage on some rabbit sperm traits (n = 0.24).

	TREATMENT						RMSE
	Control		Chitosan		rrβNGFch		
Time of storage	30′	2 h	30′	2 h	30′	2 h	
Live cells (%)	71.59 b	69.80 a	72.12 b	69.37 a	73.06 b	69.23 a	4.38
Motility rate (%)	82.5 b	78.0 a	88.1 b	71.5 a	84.8 b	70.5 a	9.90
VCL (µm/s)	95.5 b	84.4 a	101.4 b	91.9 a	103.5 b	89.5 a	10.61
LIN (%)	64.6	57.1	58.8	47.7	58.2	50.1	12.63
ALH (µm)	3.08	3.02	3.41	2.95	3.47	2.99	0.35
Apoptotic sperm (%)	6.31 A	7.87 B	6.17 A	7.68 B	5.79 A	7.16 B	0.66
Capacitated sperm (%)	5.25 a	9.18 b	5.50 a	9.4 b	12.31 c	14.06 d	2.70
Acrosome-reacted sperm (%)	5.62 a	7.875 b	6.25 a	7.75 b	11.50 c	10.43 d	1.20
p75NTR (%)	26.07 a	29.86 b	26.12 a	29.87 b	29.51 a	31.09 b	2.35
TrKA (%)	91.28 b	88.80 a	91.37 b	88.87 a	92.46 b	88.80 a	4.01
JNK (a.u.)	135.4 b	110.2 a	132.1 b	100.7 a	159.3 b	89.4 a	75.68
AKT (a.u.)	172.3 b	103.1 a	171.1 b	92.1 a	213.8 b	95.8 a	84.62
ERK 1/2 (a.u.)	0.18 a	0.15 a	0.20 a	0.15 a	0.52 b	0.41 b	0.08

Different superscript letters (a–d) in the same row indicate statistically significant differences between treatments or time points (*p* < 0.05); capital letters (A, B) indicate statistically significant differences at *p* < 0.01; RMSE: Root Mean Square Error; a.u.: arbitrary units.

**Table 2:** Correlation coefficients between molecular pathways and capacitation, acrosome reaction, and apoptosis.

	JNK	ERK 1/2	AKT	p75NTR	Capacitated Sperm	Acrosome-Reacted Sperm
ERK 1/2	0.126					
AKT	0.422 *	0.254				
p75NTR	−0.091	0.076	−0.161			
Capacitated sperm	−0.228	0.429 *	−0.134	0.405 *		
Acrosome-reacted sperm	−0.072	0.515 **	−0.055	0.360 *	0.647 **	
Apoptotic sperm	−0.184	0.396 *	−0.218	0.842 **	0.374 *	0.354 *

* *p* < 0.05; ** *p* < 0.01.

## Data Availability

The data supporting the results of this study are not publicly available, but may be obtained from the authors upon reasonable request.

## Supplementary Materials

The following supporting information can be downloaded at: https://www.mdpi.com/article/10.3390/biology14080974/s1.
