# Supplementary material for: Effect of Recombinant NGF Encapsulated in Chitosan on Rabbit Sperm Traits and Main Metabolic Pathways
Source: Biology (Basel). 2025 Aug 1;14(8):974. doi: 10.3390/biology14080974 (PMC12383564; doi:10.3390/biology14080974)
Supplement: Supplementary file 1 [file biology-14-00974-s001.zip › biology-3747780-supplementary.pdf]

Blot Figure 1

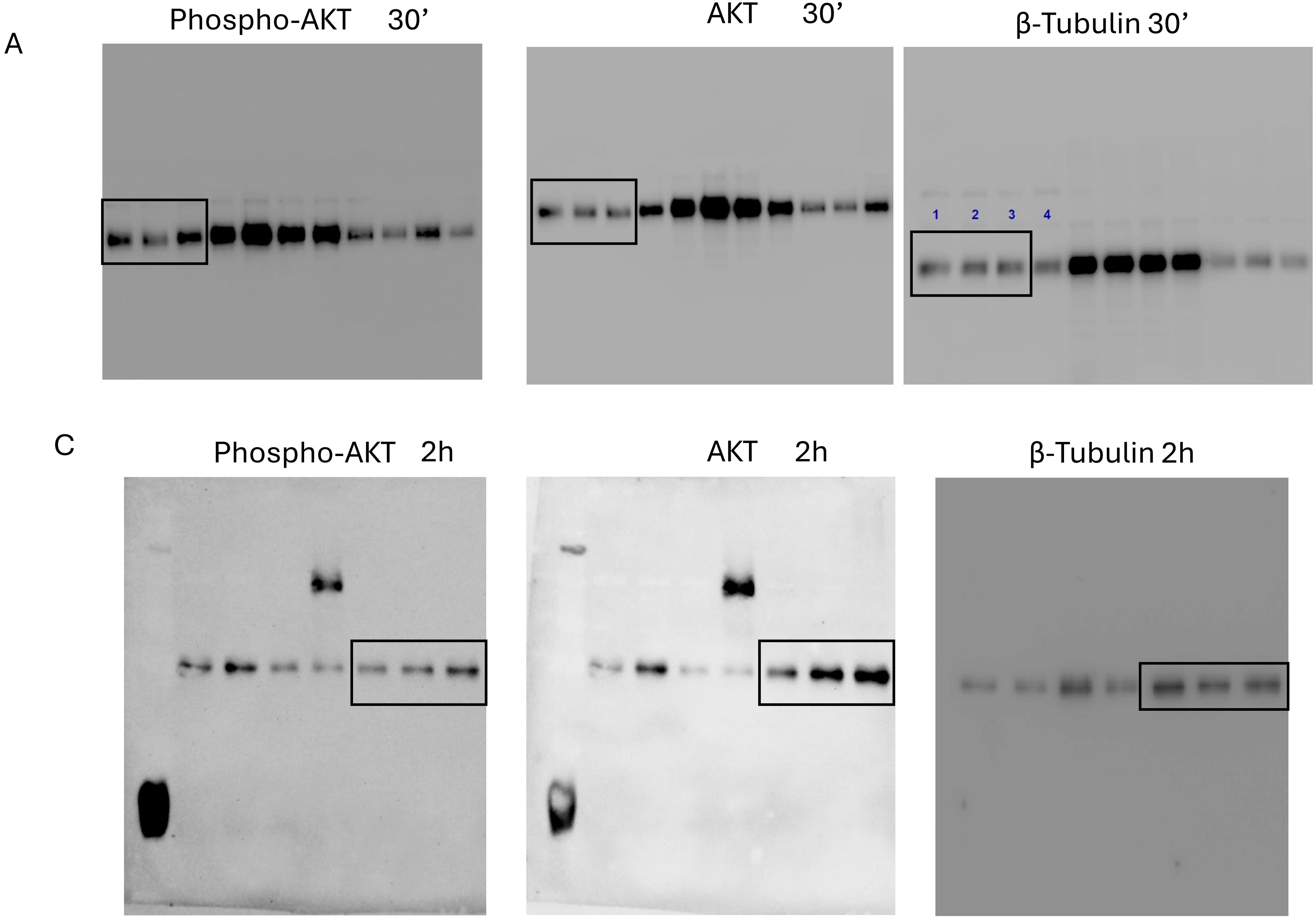

Blot Figure 2A

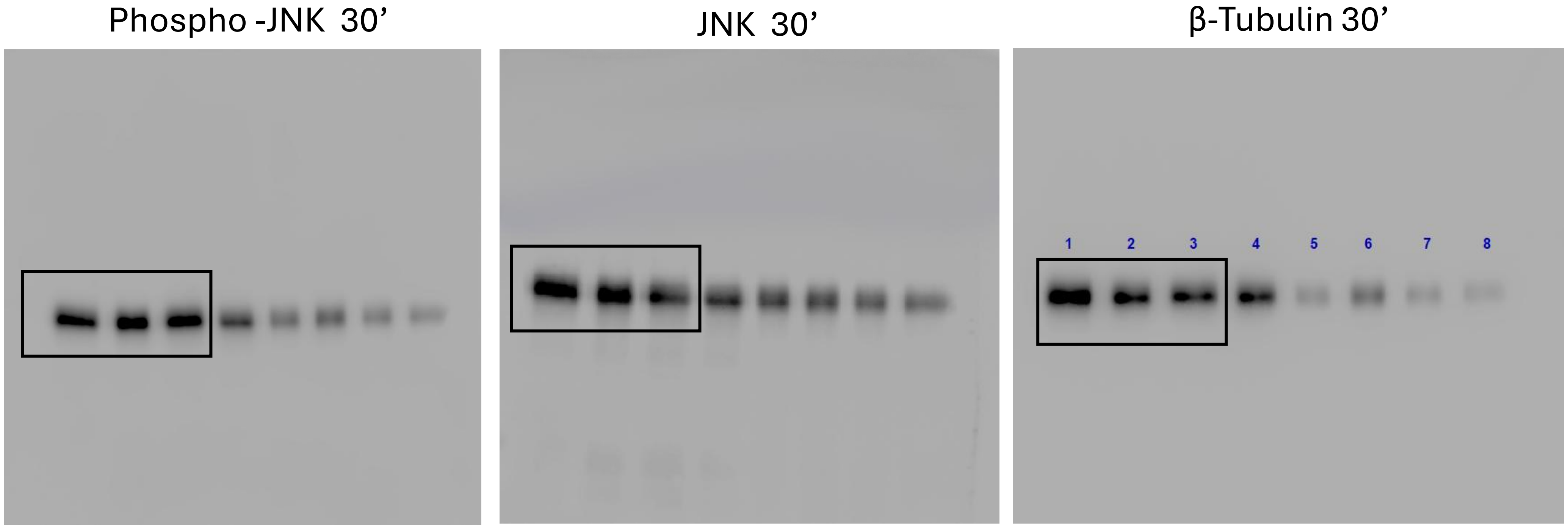

Blot Figure 2C

Phospho -JNK 2h

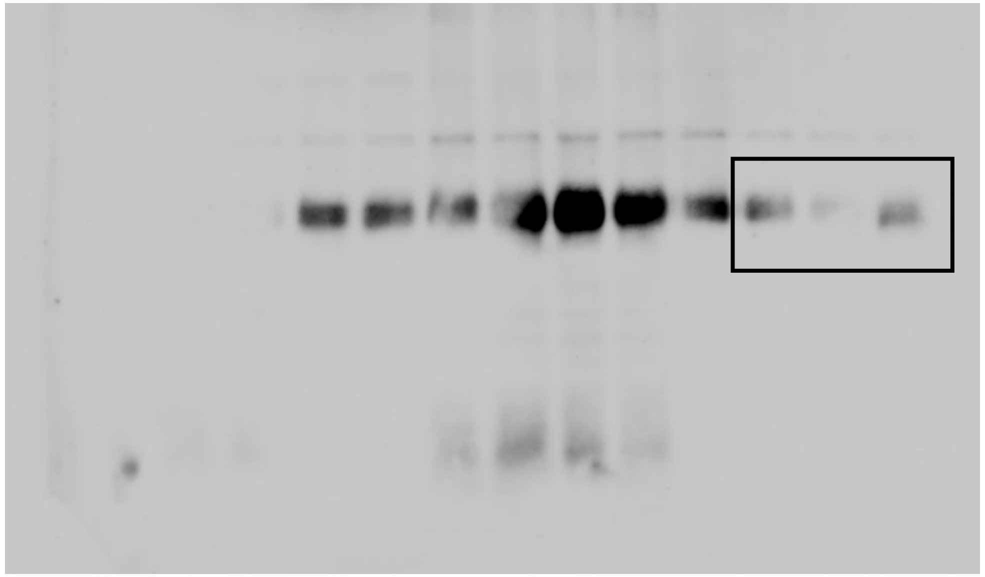

JNK 2h

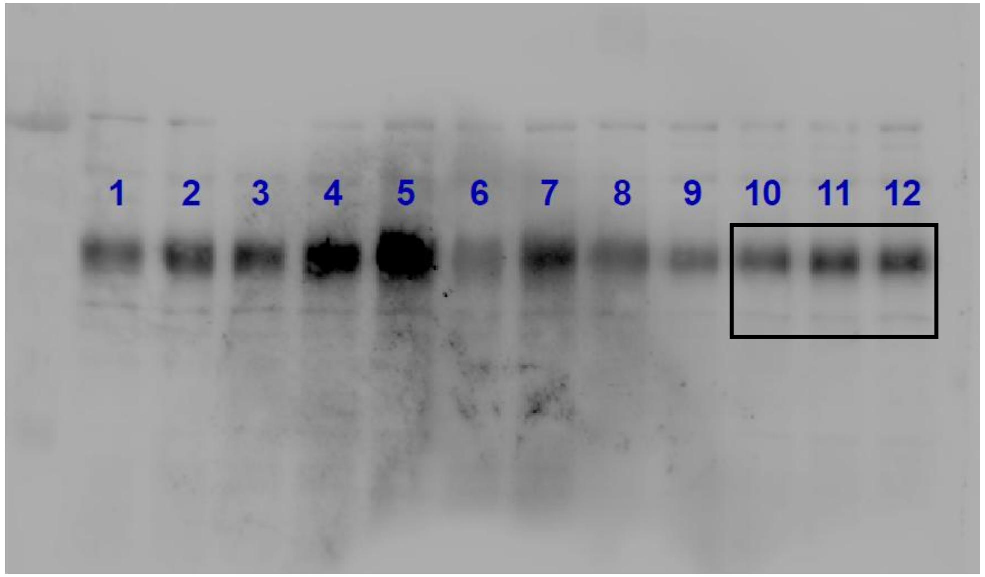

β-Tubulin 2h

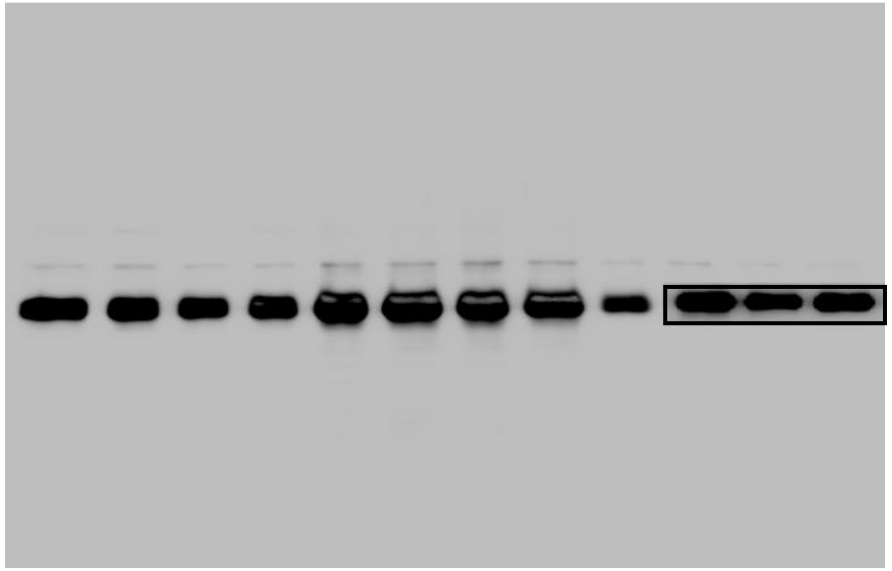

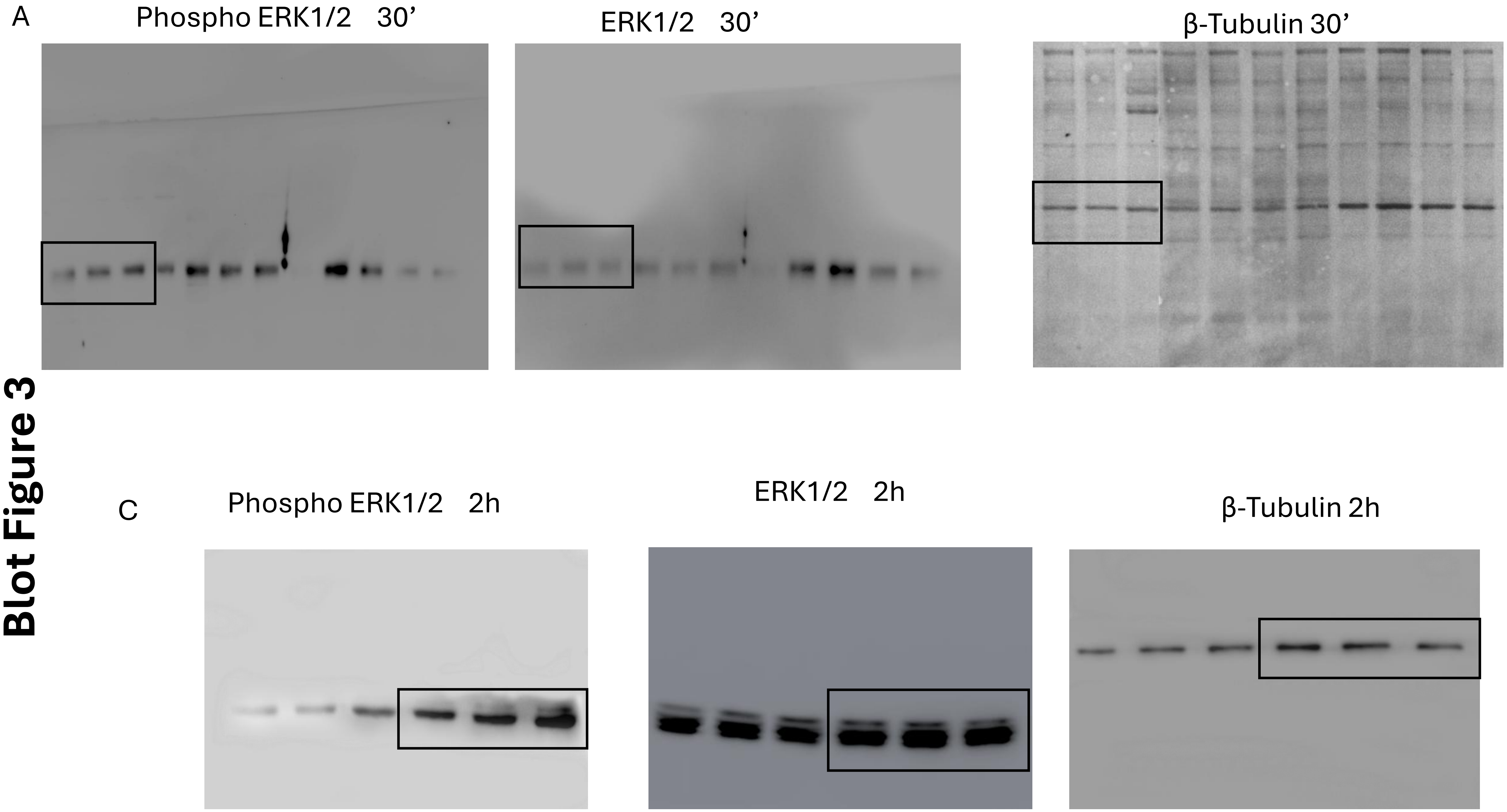

Blot Figure 3
